# How Does the VSG Coat of Bloodstream Form African Trypanosomes Interact with External Proteins?

**DOI:** 10.1371/journal.ppat.1005259

**Published:** 2015-12-31

**Authors:** Angela Schwede, Olivia J. S. Macleod, Paula MacGregor, Mark Carrington

**Affiliations:** Department of Biochemistry, University of Cambridge, Cambridge, United Kingdom; Boston College, UNITED STATES

## Abstract

Variations on the statement “the variant surface glycoprotein (VSG) coat that covers the external face of the mammalian bloodstream form of *Trypanosoma brucei* acts a physical barrier” appear regularly in research articles and reviews. The concept of the impenetrable VSG coat is an attractive one, as it provides a clear model for understanding how a trypanosome population persists; each successive VSG protects the plasma membrane and is immunologically distinct from previous VSGs. What is the evidence that the VSG coat is an impenetrable barrier, and how do antibodies and other extracellular proteins interact with it? In this review, the nature of the extracellular surface of the bloodstream form trypanosome is described, and past experiments that investigated binding of antibodies and lectins to trypanosomes are analysed using knowledge of VSG sequence and structure that was unavailable when the experiments were performed. Epitopes for some VSG monoclonal antibodies are mapped as far as possible from previous experimental data, onto models of VSG structures. The binding of lectins to some, but not to other, VSGs is revisited with more recent knowledge of the location and nature of N-linked oligosaccharides. The conclusions are: (i) Much of the variation observed in earlier experiments can be explained by the identity of the individual VSGs. (ii) Much of an individual VSG is accessible to antibodies, and the barrier that prevents access to the cell surface is probably at the base of the VSG N-terminal domain, approximately 5 nm from the plasma membrane. This second conclusion highlights a gap in our understanding of how the VSG coat works, as several plasma membrane proteins with large extracellular domains are very unlikely to be hidden from host antibodies by VSG.

## Author Summary

African trypanosomes have evolved two key strategies to prevent killing by the host immune response and, thus, maintain a long-term infection in a mammal. Both are based on a densely packed coat of a single protein, the variant surface glycoprotein (VSG), which covers the entire extracellular surface of the cell. The first strategy is antigenic variation, through which individual cells switch the identity of the expressed VSG at a low frequency and are selected by the host immune response. If the VSG is novel, the trypanosome proliferates, maintaining the infection; if it doesn't switch, or if the new VSG is not novel, it will be killed. In the second strategy, the VSG acts as a protective barrier, shielding the cell from innate and adaptive immune factors until there is an overwhelming titre of antibodies recognising the expressed VSG. In this review, the VSG coat is modelled, and past experiments that investigated how it protected the trypanosome are revisited using current knowledge of VSG sequence and structure. The conclusions are: (i) the identity of the individual VSGs explains early experimental variation; (ii) most of the VSG molecule is accessible to antibodies. This second conclusion highlights a gap in our understanding of how the VSG coat works, as several plasma membrane proteins with large extracellular domains are very unlikely to be hidden from host antibodies by VSG.

## The VSG Coat

VSGs are homodimers of two 50–60 kDa subunits held on the extracellular face of the plasma membrane by a glycosylphosphatidylinositol (GPI) anchor. VSGs have a large N-terminal domain of 350–400 residues and one or two small C-terminal domains of 20–40 residues each. The domains are connected to each other by flexible linkers [1–3]. The conformation of the linkers is unknown, as is their effect on the structure of the whole VSG. VSGs vary in sequence (for example, [4]), but have a conserved tertiary structure [5]. VSG molecules are free to diffuse in the plane of the membrane, and similar diffusion coefficients were obtained using the endogenous VSG coat on trypanosomes and VSG placed in the plasma membrane of mammalian cells in culture [6]. The rate of diffusion is high, similar to the rates measured for a range of other plasma membrane proteins, and equivalent to complete randomization of the VSG coat in 40 minutes [6]. The rate of diffusion provides strong evidence that there is minimal intermolecular affinity between VSG dimers, even at the high concentration present in the VSG coat. Estimates of the packing density of the VSG on the extracellular face of the plasma membrane have been derived from (i) measurements of the VSG copy number and estimates of the surface area (5.7 x 10^6^ VSG dimers and 180 μm^2^ [7]), and (ii) direct measurements of the cell surface area and percentage of VSG on the extracellular face of the plasma membrane (145 μm^2^ and 89% [8]). Thus, the estimated area available to each VSG dimer on the cell surface is between approximately 28 nm^2^ (cell surface 145 μm^2^) and 35 nm^2^ (cell surface 180 μm^2^), using the estimated VSG copy number above. It is worth noting that the first of the values for cell surface area was measured on cells grown in rodents, whereas the second was derived from trypanosomes grown in culture, and the discrepancy between the two values may represent a real difference due to growth conditions.

**Fig 1:**
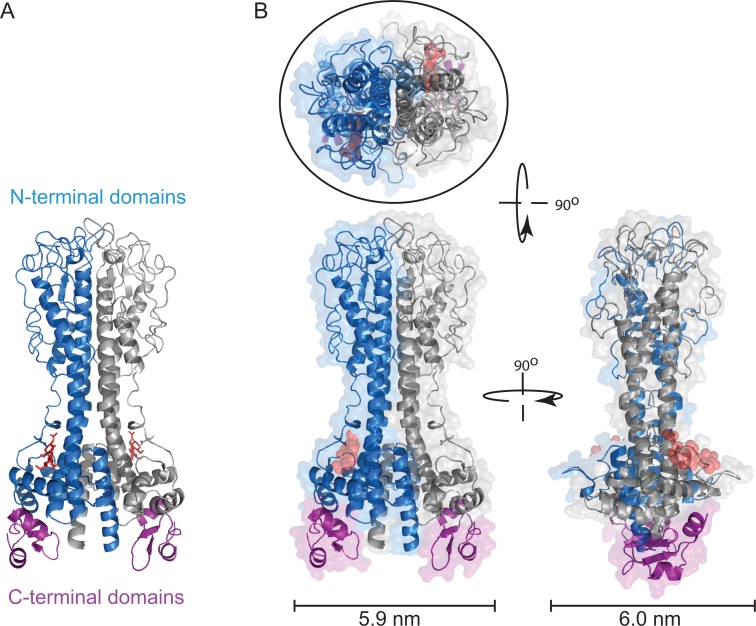
Structure of VSG221 (MITat1.2). (A) Illustrated model of VSG221 dimer showing the structures of the N-terminal domain, one monomer in blue and one in grey, and the two C-terminal domains in purple (PDB: 1VSG and 1XU6) [1,9]. The N-linked oligosaccharide in the N-terminal domain is shown in red. Three residues are shown that form the core; there are between one and three further residues not shown. The relative positions of the N- and C-terminal domains are not known, and this illustration is a model [1]. (B) Space-filling model of VSG221 viewed from the *x*-, *y*-, and *z*-axes. The maximum width dimensions of the VSG are shown below, and the fit of an ellipse of approximately 28 Å^2^ is shown around the structure of a VSG viewed from outside the cell.

The size of a VSG dimer can be derived from the structure of the N-terminal domain [5,9], and it is assumed that the long axis is perpendicular to the plasma membrane surface (Fig 1). The area taken up by each dimer can be approximated to a circle with an area of 28 nm^2^ [8], but note that this size estimate does not take into account any coordinated water molecules. This value is remarkably close to the estimates of the area available per VSG dimer as discussed above, strongly supporting the model that the vast majority of the plasma membrane is physically occluded by VSG. The VSG N-terminal domain has a long axis of approximately 10 nm, measured from the structure; allowing for some increase due to the C-terminal domain and GPI anchor, the thickness of the VSG coat is probably 12–15 nm. This value is in agreement with measurements from electron microscopy [10].

One conclusion that can be drawn from these estimates is that a significantly increased level of cell surface VSG can only occur if linked to an increase in cell surface area (for example, [11]). However, the estimates are not sufficiently accurate to distinguish between a model in which VSGs are always closely contacted by surrounding VSGs, forming a coat resembling a bubble raft [12], or whether there is a restricted amount of unoccupied space due to small variations in VSG-to-VSG distance.

## The Roles of VSG N-linked Oligosaccharides

VSG N-linked oligosaccharides have several potential functions: (i) to provide a substrate for the unfolded glycoprotein glucosyltransferase (UGGT) that catalyses the addition of glucose to a terminal mannose on an incompletely folded protein, and where export from the endoplasmic reticulum (ER) does not occur until after the glucose has been removed, reflecting a folded state for the VSG; (ii) to act as a structural element of an individual VSG; and (iii) to act as a structural element of the VSG coat. In *Trypanosoma brucei*, there are two oligosaccharyltransferases (OSTs) that function in the bloodstream form: OST1 and OST2. OST1 recognises an N-linked glycosylation site in a low isoelectric point (pI) context (five residues on either side of the N-X-S/T signal) and adds a paucimannose oligosaccharide that can subsequently act as a substrate for UGGT and can eventually be further modified by trimming down to three mannose residues and, sometimes further, through the addition of an N-acetyl glucosamine and galactose decorations. OST2 adds an oligomannose structure in response to an N-linked glycosylation site in a high pI context, which can be processed by trimming [13–15]. The specificities of the OSTs are overlapping, so an N-linked site in a neutral pI context could receive either oligosaccharide.

There is probably not a single fixed role for N-linked oligosaccharides in VSG function. Table 1 is an analysis of 33 distinctly expressed VSGs with A-type N-terminal domains, and shows length in residues and number, location, and nature of N-linked glycosylation sites, which are all features that will contribute to the overall dimensions of the VSG. There is an inverse correlation (R = -0.63) between the number of residues in the N-terminal domain and in the C-terminal domain, indicating that there may be an upper and lower limit on the number of residues for a functional VSG (S1A Fig). In contrast, there is only a very weak correlation (R = -0.11) between the molecular weight of the VSG and the number of N-linked sites (S1B Fig), suggesting that N-linked oligosaccharides do not normally have a role in increasing the size of VSGs, but do have a role other than structural in many cases, probably as substrates for UGGT. This view is supported by two other observations: (i) A small number of VSGs have no N-linked glycosylation sites, and so N-linked oligosaccharides can have no role in forming an effective coat. (ii) The majority of N-linked sites are in a low pI context (S2 Fig), and so will tend to have paucimannose glycans available for UGGT rather than the larger oligomannose glycans that might be more suitable for a space-filling role. If the role of the N-linked sites in most VSGs is to allow monitoring of folding, then it would follow that VSGs that fold efficiently no longer require such a site, whereas others that require reiterative folding cycles have retained one (or possibly more) sites. As such, the presence of an N-linked site could be more indicative of folding efficiency, rather than an element in forming a barrier. However, VSGs use every trick, and in some VSGs the oligosaccharide probably functions as a structural element in the barrier. The first example in which this might occur is in the minority of VSGs with multiple N-linked glycosylation sites in the N-terminal domain (Table 1), such as modelled for VSG118 [3], where the N-linked oligosaccharrides can occupy space between VSGs. However, it should be noted that in the one VSG with a known structure containing N-linked sugars, the oligosaccharide is held close to the VSG core and acts as a structural element in the molecules (Fig 1) [5,9]. A second example may be one N-linked glycosylation site location in VSG N-terminal domains, between residues 100 and 125, located at the plasma membrane distal tip of the VSG, where there appears to be selection for very high pI addition sites that would be almost exclusive addition of oligomannose (S2 Fig). A large oligosaccharide in this location could well affect access of external proteins.

**Table 1:** Size and N-linked glycosylation sites in VSGs with an A-type N-terminal domain.

								site 1		site 2		site 3					site 1			site 2		
		N-terminal domain	C-terminal domain	length of mature VSG	kDa mature VSG	length of A-type NTD	number of N-X-S/T	N-X-S/T location	pI	N-X-S/T location	pI	N-X-S/T location	pI	predicted Con A binding to live cells	actual Con A binding where determined	length of CTD	N-X-S/T location	N-X-S/T distance from C-terminus	pI	N-X-S/T location	N-X-S/T distance from C-terminus	pI
AnTat 1.10	K00397	A	1	451	48.0	347	2							N		104	390	-61	**3.7**	403	-48	**9.1**
IL3927 clone BV14-01/8B	AF317926	A	1	439	46.5	350	2	57	**8.8**					Y		89	362	-77	**8.2**			** **
IL3927 clone BV14-13/6	AF317920	A	1	466	50.7	375	2	130	**11.7**	266	**3.9**			Y		91						** **
IL3927 clone BV14-24/7B	AF317924	A	1	451	49.1	361	3	193	**5.2**	231	**6.7**			Y		90	403	-48	**10.0**			** **
ILTat 1.24	X56767	A	1	468	50.2	378	1							N		90	420	-48	**9.8**			** **
ILTat 1.3	J01221	A	1	455	49.4	365	2							N		90	394	-61	**3.6**	407	-48	**9.9**
LouTat 1.?	X56643	A	1	459	49.2	369	3	263	**6.2**					Y		90	398	-61	**3.6**	411	-48	**9.6**
MITat 1.12	AY935577	A	1	459	48.6	385	2	60	**5.9**	222	**8.4**			Y		74						** **
MITat 1.4	X01387	A	1	470	50.2	378	1							N		92	420	-50	**9.9**			** **
MITat 1.6	X56764	A	1	482	52.3	391	1							N	N	91	432	-50	**9.9**			** **
MITat 1.8	AY935574	A	1	419	45.0	347	1	58	**3.6**					N		72						** **
Tbg unnamed	M62629	A	1	449	47.2	375	1	350	**3.7**					N		74						** **
WaTat 1.1	M83694	A	1	461	49.1	372	2	266	**4.5**					N		89	401	-60	**4.6**			** **
BoTaR1 VSG1	X60228	A	2	450	47.4	378	2	57	**4.5**					N	N	72	446	-4	**8.8**			** **
BoTaR1 VSG20bis	X55534	A	2	438	45.7	386	4	59	**8.8**	130	**4.0**	242	**8.8**	Y		52	434	-4	**8.8**			** **
Bug 1	AJ560648	A	3	477	51.2	372	2	272	**5.8**					N		105	393	-84	**4.7**			** **
Buteba 2	AJ937312	A	2	441	46.9	379	1							N		62	437	-4	**10.0**			** **
Buteba 4	AJ937321	A	2	446	47.6	374	3	53	**4.0**	146	**6.0**			N		72	442	-4	**5.8**			** **
Buw 2	AJ937328	A	2	436	46.5	373	0							N		63						** **
IL3298	AF317916	A	2	436	47.5	373	0							N		63						** **
IL3927 clone BV14-24/7A	AF317923	A	2	438	46.8	381	3	60	**8.6**	148	**5.6**			Y		57	434	-4	**5.5**			** **
IL3960 clone BV33-01/8A	AF317929	A	2	442	47.0	390	2	100	**12.0**					Y		52	438	-4	**5.5**			** **
IL3960 clone BV33-01/8B	AF317930	A	2	423	46.0	373	2	5	**4.0**					N		50	419	-4	**5.2**			** **
ILTat 1.22	X56765	A	2	444	47.7	393	2	102	**12.0**					Y		51	440	-4	**6.3**			** **
KETRI-JN394 clone 1A	AF317931	A	2	445	47.2	393	2	313	**4.4**					N		52	441	-4	**5.5**			** **
KETRI-JN394 clone 2A	AF317932	A	2	444	48.6	380	4	107	**11.7**	187	**6.7**	281	**5.2**	Y		64	440	-4	**6.0**			** **
Kinu 1	AJ937313	A	2	450	48.1	398	1							N		52	446	-4	**5.5**			** **
MITat 1.1	X56761	A	2	443	47.9	376	2	266	**3.5**					N		67	439	-4	**6.1**			** **
MITat 1.2	X56762	A	2	433	46.3	370	2	263	**4.5**					N		63	428	-5	**8.8**			** **
MITat 1.5	X56763	A	3	429	45.0	330	3	52	**6.1**	73	**3.8**	307	**6.0**	Y		99						** **
MITat 1.7	CAI77631	A	2	450	47.3	399	2	151	**4.0**					N		51	446	-4	**4.0**			** **
RoTat 1.2	AF317914	A	2	449	47.6	385	1							N		64	445	-4	**5.8**			** **
WATat 1.2	M86646	A	2	436	45.4	384	2	301	**4.3**					N		52	432	-4	**8.8**			** **

The numbering of amino acid residues is for the mature polypeptide after removal of the N-terminal signal sequence and the C-terminal GPI-anchor addition sequence.

## Non-VSG Proteins Present in the VSG Coat

The VSG coat cannot be absolutely uniform, as there are other proteins present on the extracellular face of the plasma membrane, which raises the question of how the VSG coat acts as a physical barrier to prevent access of immunoglobulins to these non-VSG proteins. The plasma membrane can be divided into three discrete areas with different non-VSG protein compositions, each separated by some form of diffusion barrier. The first is the flagellar pocket, an invagination at the base of the flagellum, where all exo- and endocytosis occurs. The second is the flagellum membrane, and the third is the cell body membrane. Various combinations of distributions have been observed for different proteins, but the mechanism of segregating a protein to one compartment but not another is not understood. Two cell surface receptors for nutrient uptake have been identified: one for transferrin [16–18] and one for haptoglobin-haemoglobin [19]. These two receptors are concentrated in the flagellar pocket, with approximately 3,000 and 300–400 copies, respectively. The density of the VSG coat in the flagellar pocket is similar to that on the rest of the plasma membrane [8], but how the VSG density is maintained in the presence of a set of receptors and many other proteins is not understood [20]. It is also not known whether the concentration of the receptors in the flagellar pocket is advantageous for nutrient uptake and/or avoidance of immunoglobulin recognition, and/or for some other unknown reason.

**Fig 2:**
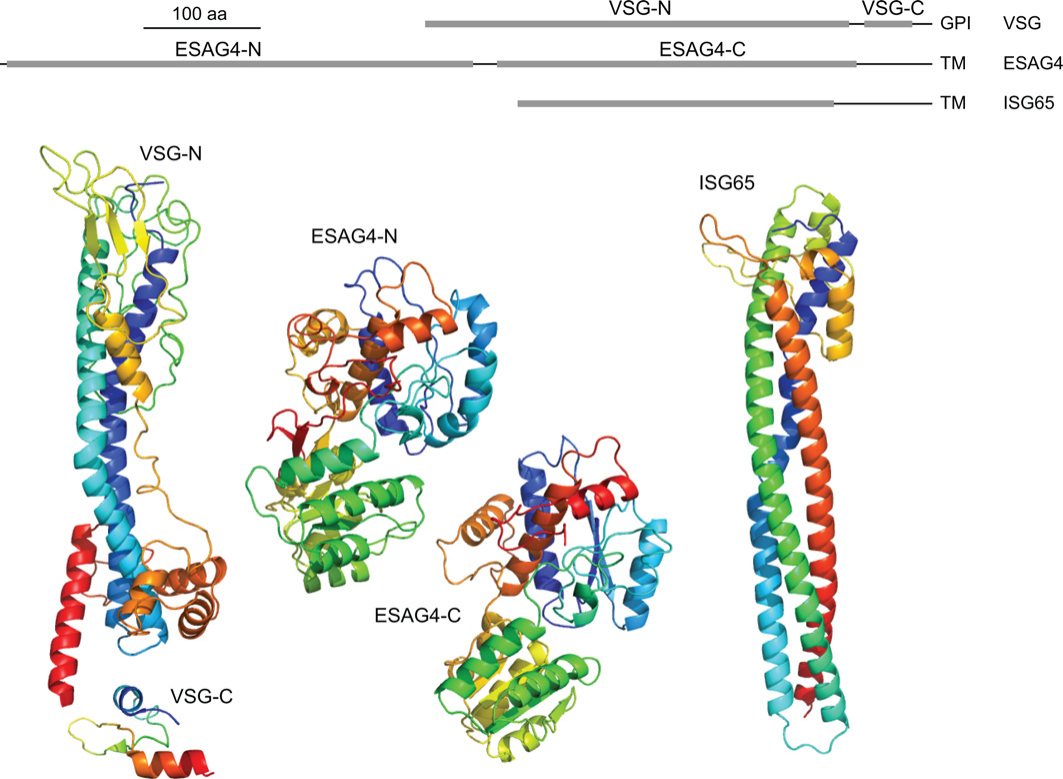
Comparison of VSG structure with modeled structures for ESAG4 and ISG65. The line diagram at the top shows the location in the extracellular part of the proteins of the real and putative structured domains. The structures below are coloured from blue at the N-terminus to red at the C-terminus. VSG structures are from PDB: 1VSG and 1XU6. The ESAG4 models were made using Phyre2 [22] and default parameters; the programme gave a 100% confidence model for both domains. The ISG65 model was made using an initial structural alignment using Fugue Profile Library Search [32], and small adjustments were made to align cysteines for disulphide bridge formation. Subsequently, Modeller was used to generate 100 models using standard Modeller scripts [31], and the model with the lowest Discrete Optimized Protein Energy (DOPE) assessment score [33] was selected to be shown here.

Many plasma membrane proteins—for example, hexose transporters—have only very small extracellular domains of less than 10 kDa, and it is likely that the VSG coat prevents access of antibodies to these domains. However, there are other proteins present on the cell body and/or flagellum plasma membrane that have extracellular domains similar in size to or even larger than the VSG. Two examples illustrate this point. First, ESAG4 and related genes encode a heterogeneous family of type 1 transmembrane proteins, some of which are localized to the plasma membrane of the flagellum. The ESAG4 family of proteins has an extracellular domain of 70–80 kDa and a cytoplasmic domain encoding an adenylate cyclase [21]. The extracellular domain is significantly larger than the VSG and can be modeled with very high confidence [22] onto a tandem di-domain of bacterial small-molecule transport proteins or substrate binding proteins (as reviewed in [23,24]). Second, the invariant surface glycoprotein (ISG) gene family also encodes type 1 transmembrane proteins localized over the whole cell surface [25–28]. ISGs have a small cytoplasmic domain and an extracellular domain that is similar size in size and structurally related to VSGs and the haptoglobin haemoglobin receptor through the use of an elaborated three-helical bundle [29,30]. ISG65 can be modeled on the haptoglobin haemoglobin receptor with a high degree of confidence, and the elongated structure has a long axis of approximately 10 nm, similar to a VSG (Fig 2) [31]. If ISGs are perpendicular to the cell surface, they would reach most or all of the way through the VSG monolayer. The copy number for individual ISGs has been estimated to between 5 x 10^4^ and 7 x 10^4^ [25,27]; if this level of expression is extended to the entire ISG family, it is likely that there are approximately 2 x 10^5^ ISGs in total, roughly equivalent to one ISG for every 50 VSG molecules. These large, non-VSG proteins pose a potential threat through immunoglobulin recognition; how these proteins avoid recognition by immune effectors remains unknown.

From the description above, there is an obvious dichotomy between two possible situations. In the most simplistic explanation, the VSG coat is structured to function by simply preventing access of host immunoglobulins to molecules such as ISGs. Alternatively, the VSG coat could function by combining a limitation on access with an active system that negates any antibody binding to proteins such as ISGs. In the context of the second model, the VSG coat is not a static entity that simply expands as the cell grows through the addition of new membrane and VSG. There is rapid endocytosis and recycling of the plasma membrane and VSG [34] that processes the equivalent to the entire cell surface every 12 minutes [35]. In addition, any VSG antibody complex that forms and protrudes above the surface of the VSG layer is subject to hydrodynamic flow resulting from movement of the trypanosome that both increases the rate of diffusion relative to uncomplexed VSG and gives the diffusion directionality [36]. The effect is to selectively force the complex toward the posterior pole, effectively concentrating it near the flagellar pocket and increasing its chances of endocytosis. It is thought that these two processes allow trypanosomes to persist as the antibody titre rises in the host until a threshold concentration is reached. The hydrodynamic flow-induced increased rate of endocytosis of surface-bound immunoglobulin does not appear to have evolved in African trypanosomes as a specialized adaptation, as it also occurs in the distantly related fish pathogen *Trypanoplasma borrelli* [37]; both might represent a specialization of an older mechanism to harvest material from the environment.

## Interactions with the Adaptive and Innate Immune System

Antigenic variation is a requirement for establishing persistent infection, as the mammalian immune system can kill trypanosomes once the immunoglobulin (Ig) titre is high enough to overwhelm the endocytosis and degradation pathway. Killing can occur through both opsonization [38] and complement-mediated mechanisms [39]. In rodent infections, near field isolates cause chronic infections lasting weeks, whereas monomorphic laboratory strains adapted for rodent growth proliferate until the rodent host dies after a few days. The difference in growth results from a loss of autoregulation of population density, leading to uncontrolled growth [40]. IgMs are important in controlling the acute infections caused by laboratory strains [41]. However, IgMs do not influence an infection when nearer field isolates are used to infect mice; the parasitaemia profile is the same in wild type and IgM-null mice [42]. This infers that the major interaction in adaptive immune system killing of trypanosomes in natural infections is probably mediated by interactions between the VSG coat and IgG. Specific VSG immunoglobulins are the mediators of clearance through the adaptive immune system, evidenced by VSG identity being the only known change in the trypanosome surface over the course of an infection. Antibodies against invariant cell surface proteins are produced during an infection but are not sufficient to produce immunity [43,44].

Binding of complement system components has also been detected. Binding of C3b and Factor B, two components of the alternative pathway C3 convertase (C3bBb), was detected after incubation in human serum [45]. Activation of the complement pathway downstream of C3 convertase did not occur, and so the trypanosomes remained viable. It is not known whether this binding is receptor-dependent and how further activation beyond C3 convertase is prevented by the trypanosome. Binding of complement C4 binding protein (C4BP), a regulatory component of both the classical and lectin pathways, has been detected, but, again, the molecular basis for the interaction has not been determined [46].

## Investigation of How the VSG Coat Functions

One way to determine how far extracellular proteins can penetrate toward the plasma membrane is to determine which structural features of the VSG are accessible on living trypanosomes. The proteins used have included: (i) VSG monoclonal antibodies (MoAbs), (ii) VSG monoclonal single domain antibodies (nanobodies, NAbs), (iii) polyclonal antisera recognising ISGs, (iv) lectins (in particular, Concanavalin A [Con A]), and (v) trypsin. Below, the results using each of these approaches are discussed, and some are re-evaluated in the light of more recent understanding of VSG structure to see how they illuminate the interaction between the trypanosome cell surface and molecules of the adaptive immune response.

## VSG Monoclonal Antibodies

There are several reports on the production of anti-VSG MoAbs and analysis of their binding to live trypanosomes [47–54]; some details and the results are summarized in Table 2. The fraction of the MoAbs that bind live trypanosomes in different reports ranges from two out of 20 to nine out of nine. This variation is probably a result of the different methods used in the initial screen to identify VSG MoAbs, as the majority of laboratories did not use binding to live cells. A second difficulty in interpretation is the requirement to take great care to perform live cell binding experiments at <4°C throughout to prevent localisation of the VSG antibody complex to the flagellar pocket and subsequent endocytosis [35]. This requirement may explain why one report found seven out of 30 VSG MoAbs localised to the flagellar pocket ([48] and Table 2). Pooling the experiments shown in Table 1, 43 out of 92 VSG MoAbs bind live cells. There are two observations that arise from these data: First, there are epitopes that are not accessible to antibodies—an observation consistent with dense VSG packing causing restricted access. Second, externally accessible epitopes are not a small percentage of the total number of epitopes.

**Table 2:** Summary of VSG MoAb experiments.

VSG	MoAb numbers; immunogen	Screen to select MoAb	Fixed cell binding	Live cell binding	Epitope groups	Refs
WaTat 1.1	Seven MoAbs; irradiated trypanosomes or purified VSG	purified VSG RIA	ND	Five from seven	Seven surface, six buried, three flagellar pocket	[47]
WRATat 1	30 MoAbs; purified VSG (?)	acetone fixed blood smears	30 from 30	14 out of 30 plus seven out of 30 to flagellar pocket	Seven surface, six buried, three flagellar pocket	[48]
MITat 1.6	Nine MoAbs; infection and cure	purified VSG RIA	Nine from nine	Two from nine	One surface, four not exposed	[49,50]
DiTat 1.3	20 MoAbs; infection and cure	immunofluorescence ethanol fixed	20 from 20 ethanol fixed Ten from 20 on paraformaldehyde fixed	Two from 20	One exposed (?)	[51]
MITat 1.2	Three MoAbs; infection and cure	purified VSG ELISA and/or live cell binding	16 from 16	Three out of three infection and cure	Eight surface, one buried	[52]
	Five MoAbs; purified VSG			Zero out of five MoAbs purified VSG		
	Eight MoAbs; X-irradiated trypanosomes			Seven out of eight MoAbs X-irradiated trypanosomes		
VSG78	Seven MoAbs; infection and cure	neutralization immunofluorescence acetone fixed purified VSG ELISA	Seven from nine	Nine from nine based on neutralisation	Identified five residues that, when mutated, conferred protection against neutralisation by anti-78 MoAb. All located on top of VSG.	[53]
MITat 1.4	One MoAb; trypanosomes freeze thawed in the presence of Zn2+	paraformaldehyde fixed plus binding to live cells at 0°C plus western blot	One from one	One from one	One exposed on side of VSG	[54]

It is commonly believed that MoAbs observed to bind fixed trypanosomes but not live cells result from a disruption of the surface coat during the fixation process and a concomitant exposure of epitopes inaccessible in live cells. There is a further consideration that must be made to explain the increased accessibility of some MoAbs to VSG in fixed cells or in vitro (ELISA/western blot) but not in vivo. Denaturation of the VSG will expose epitopes normally hidden by being in the huge dimerisation interface [5] and/or internal within the structure VSG. Many of the screening procedures used to select MoAbs would have resulted in complete or partial VSG denaturation, including coating plates for solid phase radioimmunoassay (RIA) or ELISA, solvent fixation, air-drying, and, possibly, formaldehyde fixation. Such MoAbs would give the appearance of recognising epitopes that were inaccessible in live cells, and no analysis of the MoAbs above was performed to determine whether they recognised epitopes only after denaturation.

**Fig 3:**
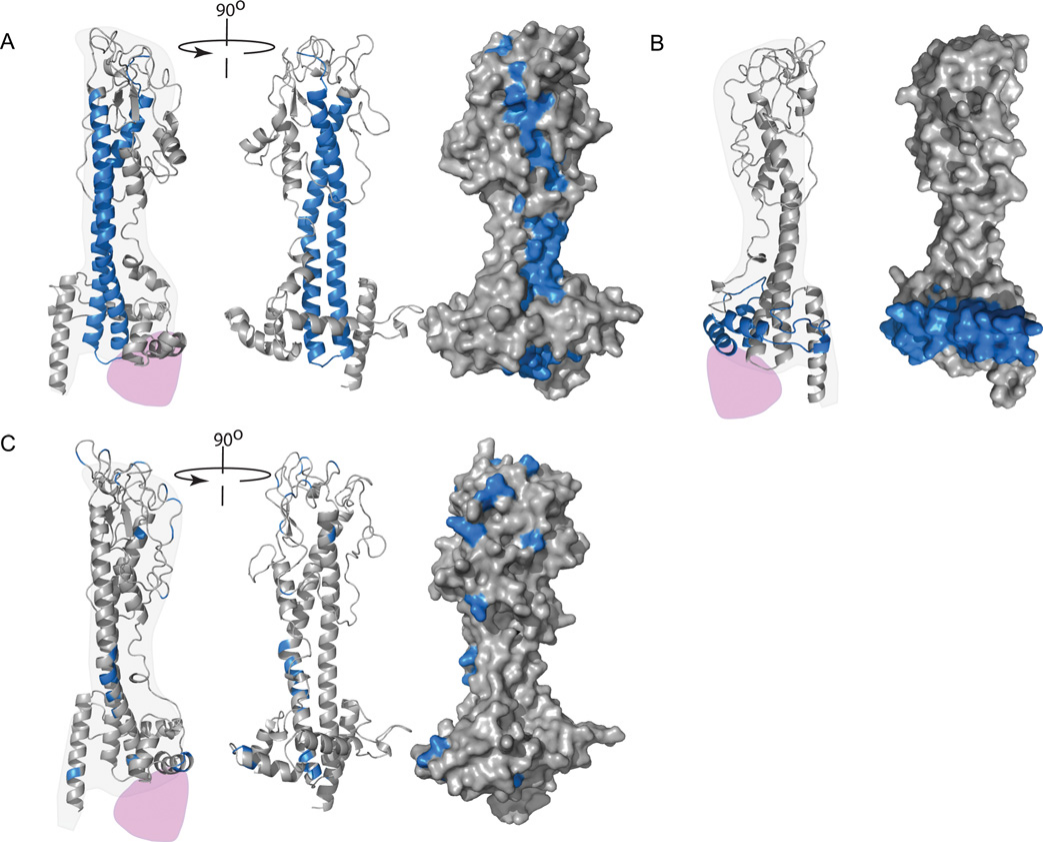
VSG models. (A) A model of VSG121 showing the location of the cyanogen bromide fragment p19 (blue) that contains the epitopes for MoAbs that bound live trypanosomes. From the left, one monomer orientated so the dimerization interface runs vertically up the page; second, rotated approximately 90° so that the dimerization interface has turned away from the observer; third, same view with the surface added. There are potential surface-exposed epitopes along the entire length of the domain. (B) A model of VSG117 showing in blue the location that contained the epitope recognised by a MoAb that bound live cells. (C) Model of VSG WATat1.1 showing the location of differences with the related VSG WATat1.12. A monoclonal antibody that recognises an epitope in WATat1.1 does not bind WATat1.12, so the epitope probably contains one of these differences. An envelope for one possible position of the C-terminal domain is shown in purple.

A set of studies mapped the epitopes recognised by MoAbs that bound live cells onto the molecular structure of the VSG [49,50,53–55]. The first analysed nine monoclonal antibodies that were screened for VSG121 binding using solid phase RIA [49,50]. All nine MoAbs bound VSG in air-dried blood smears (no other fixation), and two bound live trypanosomes in suspension. The two MoAbs that bound live cells did not bind VSG in Western blots, whereas the other seven did. The epitopes were mapped using competition RIAs between the MoAbs and sera raised against purified cyanogen bromide peptides. The two MoAbs that recognised live cells were difficult to map but were weakly competed by anti-p19, which contains residues 1 to 111 of the mature VSG. The remaining MoAbs either recognised epitopes in p16 (residues 112 to 332) or conformational epitopes containing components from both p19 and p16. Subsequent to this report, it emerged that VSG structures are conserved [5], that p19 corresponds to the coil running the entire perpendicular length of the VSG, and that p16 contains most of the N-terminal domain (Fig 3A). Thus, it is not possible to estimate a value for the penetration of the two live cell binding MoAbs into the VSG monolayer, and the remaining seven probably recognised epitopes exposed by denaturation on drying or SDS treatment.

The second study [54] went to great lengths to isolate a VSG117 MoAb that recognised VSG on both live cells and after western blotting. Unlike the analyses above, this work was performed after the structure of a VSG had been determined and exploited the conservation of structure to map the epitope using recombinant chimaeric VSGs. The epitope was mapped to a region more than halfway down the VSG N-terminal domain (Fig 3B). This set of experiments provided very strong evidence that immunoglobulin G molecules can penetrate a minimum of 6 to 8 nm into the VSG coat, and most of the surface of the VSG N-terminal domain is accessible.

In a third study [55], a panel of seven MoAbs raised against VSG WaTat1.1 were tested for binding to a second VSG, WaTat 1.12, known to cross-react with WaTat1.1 polyclonal antisera. One of the seven MoAbs did not cross-react, and, since there are only 24 point differences in the sequences of the two VSGs, it can be assumed that one of these differences occurs in the epitope. The differences are located throughout the structure of the N-terminal domain (Fig 3C). From this comparison, it is not possible to identify the epitope recognised by the single selective MoAb. It is attractive to speculate that the difference lies between residues 73 and 82, which contain seven out of 24 differences; however, these residues are largely buried in the dimerization interface and would not be accessible in the VSG dimer.

The fourth study [53] used live trypanosomes and neutralising MoAbs that recognised VSG 78 to select mutants that escaped but still expressed a VSG recognised by a polyclonal anti-VSG 78. Several monoclonal antibodies were used to recognise different conformational epitopes. Two independent clones that escaped neutralisation with the first monoclonal antibody had changed serine 192 to arginine. The sensitivity to other monoclonal antibodies remained, showing that the overall structure was not affected by the mutation. Another mutant selected with the second monoclonal antibody expressed a VSG78 where glutamine 172 was changed to glutamic acid. The last isolated mutant selected with the third antibody had several changes in the VSG gene. There was a gene conversion in the 5′ region of the ORF and, in addition, a mutation in the codon 220 that was probably responsible for the resistance phenotype. All mutations identified are located in the loops at the membrane distal end of the VSG that would be readily accessible to antibodies on live cells.

## VSG Single Domain Monoclonal Antibodies

Single domain antibodies (nanobodies, or NAbs) are derived from classes of immunogloblins that contain only two heavy chains and are unique to camelids. The variable domain is approximately 15 kDa, containing the antigen binding variable loops, and can be made as a recombinant protein. When these were produced against VSG AnTat1.1, a range of NAbs recognising different epitopes were isolated, including one that recognised the carbohydrate moiety on three different VSGs, all having N-linked oligosaccharides in the N-terminal domain [56]. The oligosaccharides on these three VSGs are located just over halfway down the N-terminal domain, and so the NAbs penetrate some distance into the VSG layer, as observed for one of the MoAbs described above [54].

While MoAb and NAb binding to the surface of the VSG N-terminal domain has been observed in multiple studies, the C-terminal domain does appear to be protected. Two polyclonal antisera to recombinant C-terminal domains both bound strongly after fixation but showed no binding to live trypanosomes [57]. This observation provides strong evidence that the VSG coat greatly reduces or eliminates penetration of immunoglobulins to the VSG C-terminal domain and, thus, the plasma membrane.

## Antibodies Recognising Invariant Surface Glycoproteins

ISG65 and ISG75 are the two best-characterised invariant proteins present over the extracellular face of the plasma membrane of the entire body [25–27,43]. As detailed above, the extracellular domains of approximately 350 and approximately 440 residues, respectively, are comparable sizes to a VSG. Modeling of the structure of the domain suggests that the ISGs have a long coil of approximately 10 nm (Fig 2) [29]. Are the ISGs accessible to antibodies on live cells? The interactions with immunoglobulins were tested in two ways: first, the binding of anti-ISG immunoglobulin to fixed and live cells was compared; second, mice were immunized with recombinant protein and challenged with a trypanosome infection. There was a discrete binding of anti-ISG75, but not anti-ISG65, to live cells in one study [43]; subsequently, however, binding of anti-ISG65 has been reported with an independent antiserum [58]. The binding of anti-ISG75 was low compared to binding after fixation and was dependent on the VSG expressed, suggesting some, but not complete, limitation on accessibility. These experiments are difficult to interpret; at a simple level they could be taken to show that ISGs are accessible, but the epitopes recognised by the antisera were not characterized, and a confirmation through the use of defined MoAbs is required.

However, any ISG accessibility does not necessarily lead to immunity. Prior immunization with ISGs provided no protection against infection in mice [43]. It is also worth noting that infected people and animals produce anti-ISGs, but these do not provide protection [44]. Together, these observations allow a tentative conclusion that ISGs can be accessed by immunoglobulins, but binding is limited and tolerated by the trypanosome. The mechanism of this tolerance is probably related to the recycling of the cell surface [35]. One model for the tolerance might be that the combination of low ISG copy number and rapid recycling time does not allow the bound immunoglobulin to trigger a response. If this is the case, the VSG would shield part, but not all, of the ISG protein, ruling out a simplistic model of complete inaccessibility to non-variant surface proteins.

## Concanavalin A

The Con A monomer is 29 kDa and, at pH 7.5 in physiological salt concentrations, is in approximately 1:1 dimer-to-tetramer equilibrium [59]. The dissociation constant for monomeric interaction is around 50 μM, with a dissociation rate of 4/s; consequently, any binding detected to live or fixed cells after washing must be multivalent. Con A is subject to very complex post-translational modification [60], and the properties of different batches of Con A are affected by different amounts of proteolysis of the monomeric units. The main effect of this variability is not on binding but on valency, with proteolysed subunits remaining as dimers [61]. Succinylated Con A is locked in the dimeric form and has been used to reduce variability in the reagent in some experiments [62]. Con A preferentially binds a branched mannose trisaccharide [63] and, in VSGs, will bind N-linked sites modified with oligomannose rather than paucimannose. The response to the pI context of the N-linked glycosylation site is gradual, and away from the extremes of pI values, many sites are modified with either paucimannose or oligomannose side chains. This means that predictions of whether a live trypanosome expressing a particular VSG will bind Con A (Table 1) have to be taken with a pinch of salt. The ability of Con A to bind to live trypanosomes was determined in many labs (for example, [62,64–66]). To summarize these results, Con A bound trypanosome clones expressing some VSGs but did not bind other clones expressing different VSGs. The majority fell into the second category, consistent with the predictions in Table 1. Nearly all these studies were performed before detailed sequence and structural data were available for VSGs. In light of what is known now, some of these data can be explained. The locations of the sites vary, as some VSGs have a single site in the C-terminal domain and others a single site in the N-terminal domain (Table 1). Obviously, a VSG with no N-linked sites will not bind Con A; for other VSGs, binding will depend on accessibility, which itself will be related to the location of the N-linked oligosaccharide on the tertiary structure of the VSG and on the context pI of the N-linked site. Most of the experiments to determine the nature of the binding of Con A to live trypanosomes were performed using VSGs of unknown sequence (no sequences were available at the time), but a number of the VSGs have been subsequently characterized. For example, trypanosomes expressing VSG MITat 1.6 (VSG 048) are not bound by Con A unless fixed or treated with trypsin [65], and this VSG was later shown to have a single N-linked glycosylation site in the linker between the two C-terminal domains [2]. The importance of the identity of the VSG in determining whether the N-linked oligosaccharide is accessible to Con A was clearly demonstrated in a study that used ten *T*. *equiperdum* clones expressing different VSGs; three were agglutinated, seven were not [64]. The sequences of one Con A binding VSG (BoTat 78) and one non-binding VSG (BoTat 1) have subsequently become available, and the location of N-linked glycosylation sites in these two VSGs provides an explanation for the difference: one site in VSG BoTat 1 is in the C-terminal domain; the other is at the base (plasma membrane proximal) of the N-terminal domain. In contrast, the sites in VSG BoTat 78 are located in the N-terminal domain, where they could present the oligosaccharides pointing toward the top of the VSG coat.

## Trypsin

Trypsin is a 23 kDa protease with specificity for lysine and arginine residues. When trypsin is added to live trypanosomes, it is able to digest VSG and release fragments from the cell ([67], for example). Different VSGs are released at different rates [67]. In terms of VSG release, the most trypsin-sensitive point is the hinge between the N- and C-terminal domains. One way to explain the variations in sensitivity to trypsin is that the enzyme is near the size limit able to gain access to the hinge part of the VSG, and some VSGs block access, whereas other do not. However, this model ignores the availability of substrate, and some VSGs may simply be better substrates than others. It would be interesting to compare the trypsin sensitivity of different VSGs comparing purified protein and live cells.

## Other African Trypanosomes

Other species of African trypanosomes are much less well understood both in terms of the repertoire of functional VSGs and in non-VSG surface proteins. The genomes of *T*. *congolense* and *T*. *vivax* have been analysed for putative surface proteins [28], but there has been limited biochemical analysis to support this. In *T*. *congolense*, VSGs do not appear to have a structured C-terminal domain(s) but do have C-terminal extension of approximately 30 residues beyond the end of the structured N-terminal domain. The effect or role of this extension on the structure of the VSG coat is not known, and it may play an equivalent role to the C-terminal domain in *T*. *brucei* VSGs. *T*. *vivax* VSGs do not have any significant polypeptide extension on the C-terminus after the end of the structured VSG N-terminal domain, and there is little knowledge of experimentally identified non-VSG surface proteins.

## Conclusions

The molecular detail of how the VSG coat negates the adaptive immune response is interesting in itself, but is also relevant to identifying therapeutic strategies. One important question to be answered is how far extracellular macromolecules can penetrate into the VSG coat. The answer to this question will provide information on the effectiveness of the VSG coat as a physical barrier and whether the cell has evolved systems to overcome immunoglobulin binding to lower copy number invariant antigens.

**Fig 4:**
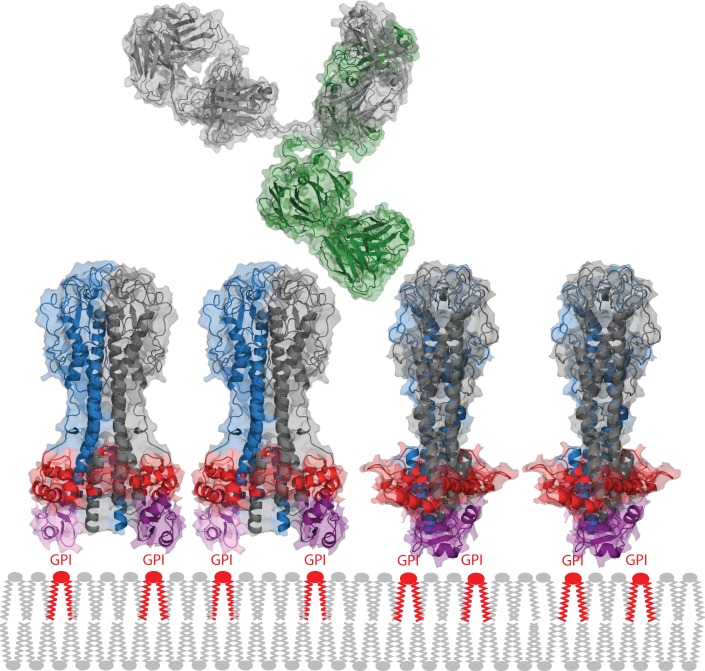
Packing of the VSG on the plasma membrane and comparison with an immunoglobulin G. The VSG spacing is based on experimentally determined copy number and surface area estimates. The widest part of the VSG N-terminal domain is shown in red and the C-terminal domain in purple. Together, these two features probably form the barrier that restricts access of immunoglobulins to the plasma membrane. One heavy and one light chain in the IgG is shown in green, the other pair in grey. The VSG structure is derived from PDB 1VSG and 1XU6, the IgG from PDB 1IGY.

It is hard to draw many firm conclusions from the existing data, primarily due the absence of defined structures, ligands, and ligand binding sites. As examples: (i) The structure of ISG65 is only a model, and the epitopes recognised by the polyclonal ISG antisera have not been characterized. (ii) The structures of the N-linked oligosaccharides for some VSGs have been solved, but their location within the VSG coat is unknown (although it has been modeled [3]), and the relative affinity of Con A for the different N-linked oligosaccharides has not been determined. Another problem is that most of the data were collected before sequencing of VSGs became routine. Even now, only a subset of the experiments can be looked at with the sequence in one hand and structure-based hindsight in the other. The experiments with unambiguous data on the penetration of a macromolecule into the VSG coat provide very strong evidence that an intact immunoglobulin G could reach the lower part of the VSG N-terminal domain [54]. It is probable that the base of the VSG N-terminal domain, the region with the largest cross-sectional area perpendicular to the cell surface, represents the real physical barrier guarding the plasma membrane (Fig 4). It is possible that the C-terminal domain reinforces this barrier, as shown in Fig 4, but this location remains a model.

## Supporting Information

S1 Fig (A) Plot of C-terminal domain length against N-terminal domain length for 33 VSGs with an A-type N-terminal domain (Table 1). The boundary between the domains was defined as 10 residues to the n-terminal side of the first cysteine in the C-terminal domain. This location approximates to the middle of the linker between the two domains. R is the correlation coefficient. (B) Plot of VSG molecular weight based on amino acid sequence alone against the number of N-linked glycosylation sites. R is the correlation coefficient.
(PDF)

S2 Fig Plot of the pI of the N-linked glycosylation site context against location in the primary structure for the N-terminal domains of the 33 VSGs in Table 1. The structure of VSG221 is shown to the left, and secondary structure regions are highlighted in colours both in the structure and below the *x*-axis. The shading indicates the tendency for the addition of oligomannose at high pI to paucimannose at low pI.
(PDF)
