# Supplementary figures and images for: How Does the VSG Coat of Bloodstream Form African Trypanosomes Interact with External Proteins?
Source: PLoS Pathog. 2015 Dec 31;11(12):e1005259. doi: 10.1371/journal.ppat.1005259 (PMC4697842; doi:10.1371/journal.ppat.1005259)

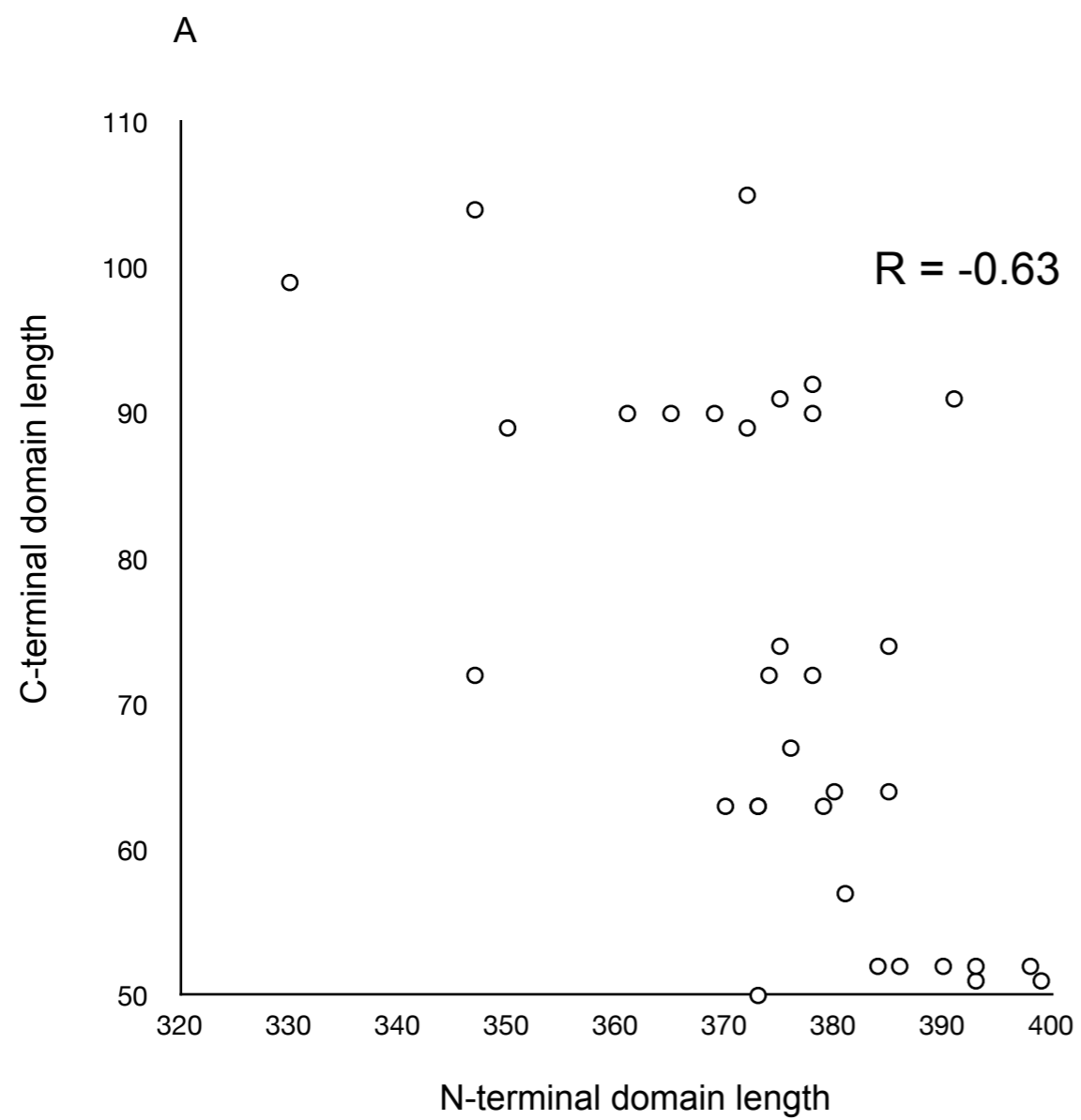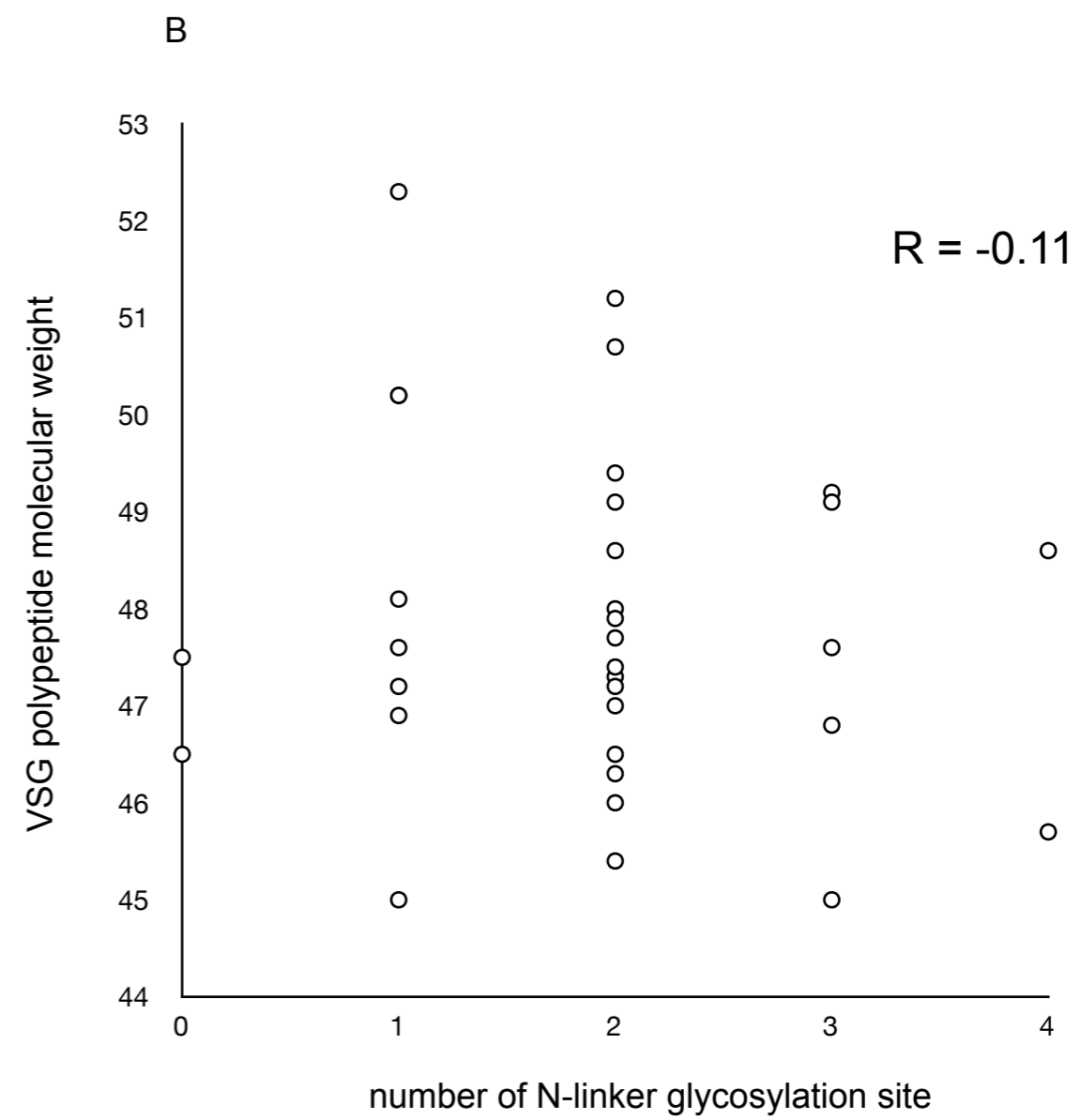

Supplementary Figure 1

Supplement: S1 Fig — R is the correlation coefficient. (PDF) [file ppat.1005259.s001.pdf]

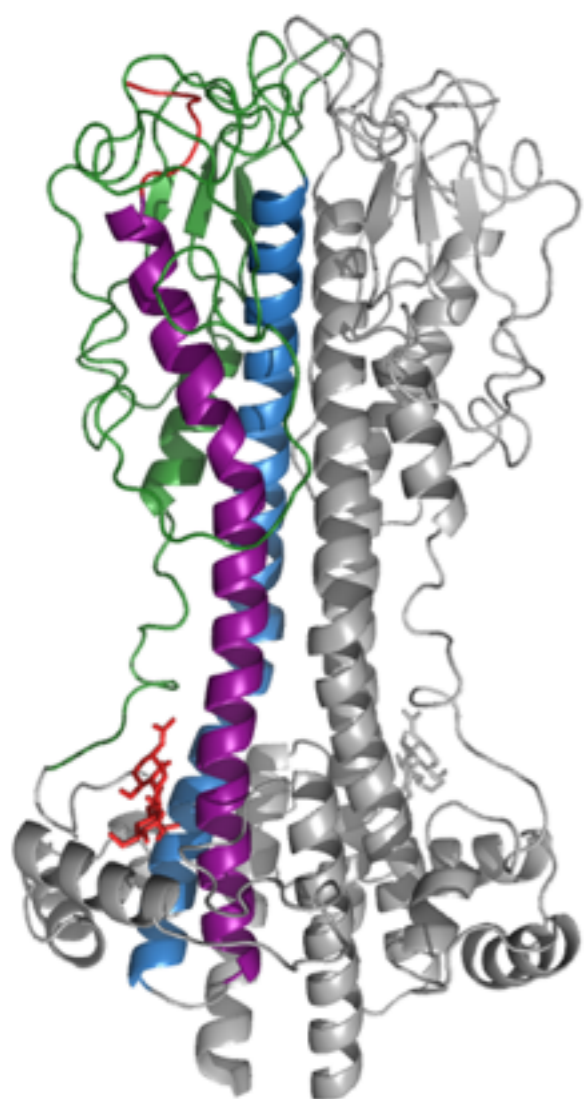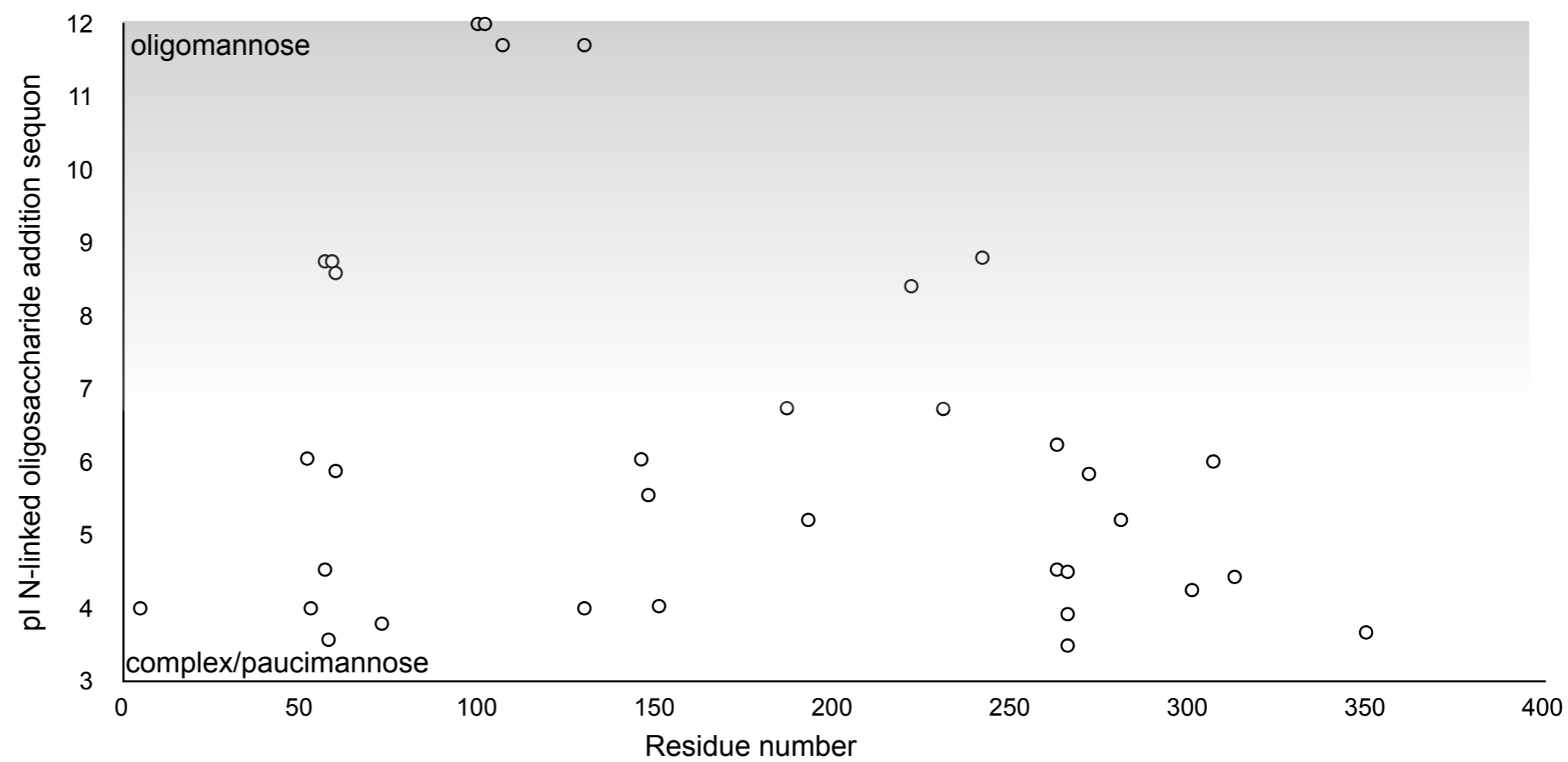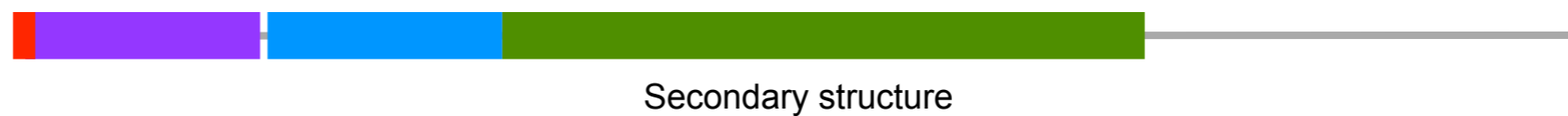

Supplementary Figure 2

Supplement: S2 Fig — The structure of VSG221 is shown to the left, and secondary structure regions are highlighted in colours both in the structure and below the x-axis. The shading indicates the tendency for the addition of oligomannose at high pI to paucimannose at low pI. (PDF) [file ppat.1005259.s002.pdf]
